# Refugees, trade, and FDI

**DOI:** 10.1093/oxrep/grac022

**Published:** 2022-09-15

**Authors:** Dany Bahar, Christopher Parsons, Pierre-Louis Vézina

**Affiliations:** * Brown University, USA; ** University of Western Australia, Australia; *** King’s College London, UK

**Keywords:** refugees, trade, investment, FDI, international migration, diasporas, migrant networks, F14, F21, F22, F66

## Abstract

Humanitarian policies aimed at welcoming forced migrants may yield unexpected economic dividends. This article focuses on the trade and investment links forged by refugees between their countries of resettlement and the origins they fled. We document how such immigrant-links differ in the case of refugees, focusing on why their opportunity sets might differ and the difficulties in establishing economic connections against a backdrop of civil conflict and political unrest. We conclude by discussing a range of policies aimed at engaging refugee diasporas to foster development at refugees’ origins.

## I. Introduction

Fish and chips, arguably *the* quintessential British dish, is widely attributed to Joseph Malin, an Ashkenazi Jew who fled persecution in Eastern Europe and settled in London’s East End in the nineteenth century.^1^ Malin’s ‘invention’ was to combine fried potato with fried fish. The potato element was likely brought to Britain’s shores by French protestants (Huguenots) fleeing religious persecution following King Louis XIV’s passing of the Edict of Fontainebleau.^2^ The fried fish, originally termed ‘pescado frito’ was a Sephardic Jewish dish commonly served on Shabbat in Spain and Portugal (Marks, 1999), one ultimately brought to England following Jewish persecution across the Iberian peninsula.^3^ The ‘British classic’ is therefore the culmination of culinary inventions developed elsewhere and brought separately to Britain and subsequently combined into a new dish, all by persecuted minorities.

For centuries, those fleeing persecution have been discovering new frontiers, spreading ideas, diffusing knowledge, and transferring technology across the globe. Refugees constitute bridgeheads between countries; conduits between cultures; often forging links between far-flung and culturally disparate corners of the world. Prominent examples in the economics literature include: Huguenots raising productivity in Prussian textiles (Hornung, 2014) and contributing to the development of the South African wine industry (Bahar and Rapoport, 2018), and Jewish and Russian scientists revolutionizing science in the US (Moser *et al.*, 2014; Ganguli, 2015).

The focus of this paper, however, is on revisiting the more contemporaneous literature that examines the role of refugees—as identified by the sum of all populations tracked by the United Nations High Commission for Refugees (UNHCR)—in fostering international trade and cross-border investment: since it is along these dimensions that refugees have been shown to affect the development of their home countries. The Lebanese, for example, first settled in West Africa following the 1893 silk worm crisis (Khater, 1996), the pioneers of which settled in British Sierra Leone. Today, the Lebanese represent a key minority across West Africa, playing a significant role in fostering trusted trading relationships (Winder, 1962). Indeed, Baobab (2011) argues that many Lebanese in West Africa operate large businesses dominating regional trade due to their trusted networks in places far further afield, for example like China and Brazil. Humanitarian policies aimed at admitting refugees can therefore yield unintended economic dividends.

Refugees often represent new cultural additions to host nations, some of the first to experience the cultures at both origin and destination, and as such they are (potentially) ideally positioned to introduce new ideas from their homelands into their host nations and conversely to transplant newly formed ideas home. This ‘diversity dividend’ can result in refugees’ cultural perspectives proving particularly valuable (Legrain, 2016). Should a new opportunity set become available, refugees are arguably best placed to exploit it first. This fact may serve to explain, at least to some extent, the incredible success of particular individual refugees and their children.^4^

The characteristics that refugees embody, together with their experiences, may culminate in refugees proving especially entrepreneurial, the so-called ‘dynamism dividend’ (Legrain, 2016). Indeed, refugees have been determined to be the most entrepreneurial of all migrants in Australia, while also owning the highest proportion of self-owned unincorporated businesses.^5^ Similarly, in the UK, asylum seekers, conditional on being employed, are six percentage points more likely to be self-employed relative to the UK-born (Kone *et al.*, 2021).^6^ In the United States, Dagnelie *et al.* (2019) show that refugees are more likely to be employed within 90 days of arrival should more business owners (from the same origin) feature in their networks, but less likely to be employed should more employees be represented in their network.^7^

The circumstances of refugee departures are often fraught with danger, however. Chin and Cortes (2015) highlight that 47 per cent of US refugee claims have been subject to persecution, as opposed to just 4 per cent among non-refugee applications. When departing Vietnam for example, the UNHCR estimate that between 200,000 and 400,000 Boat People perished at sea (Vo, 2006). Relatedly, the strife that refugees left behind likely undermines the functioning of the home market, one that is already likely characterized by weak institutions. While economic theory suggests that the documented ability of migrants to foster trade and investment across the globe might also translate to refugees, concurrently, political strife and the absence of the rule of law at refugees’ origin might nevertheless conspire to prevent such links being established due to non-functioning or ill-performing markets (Anderson and Marcouiller, 2002).

**Figure 1:**
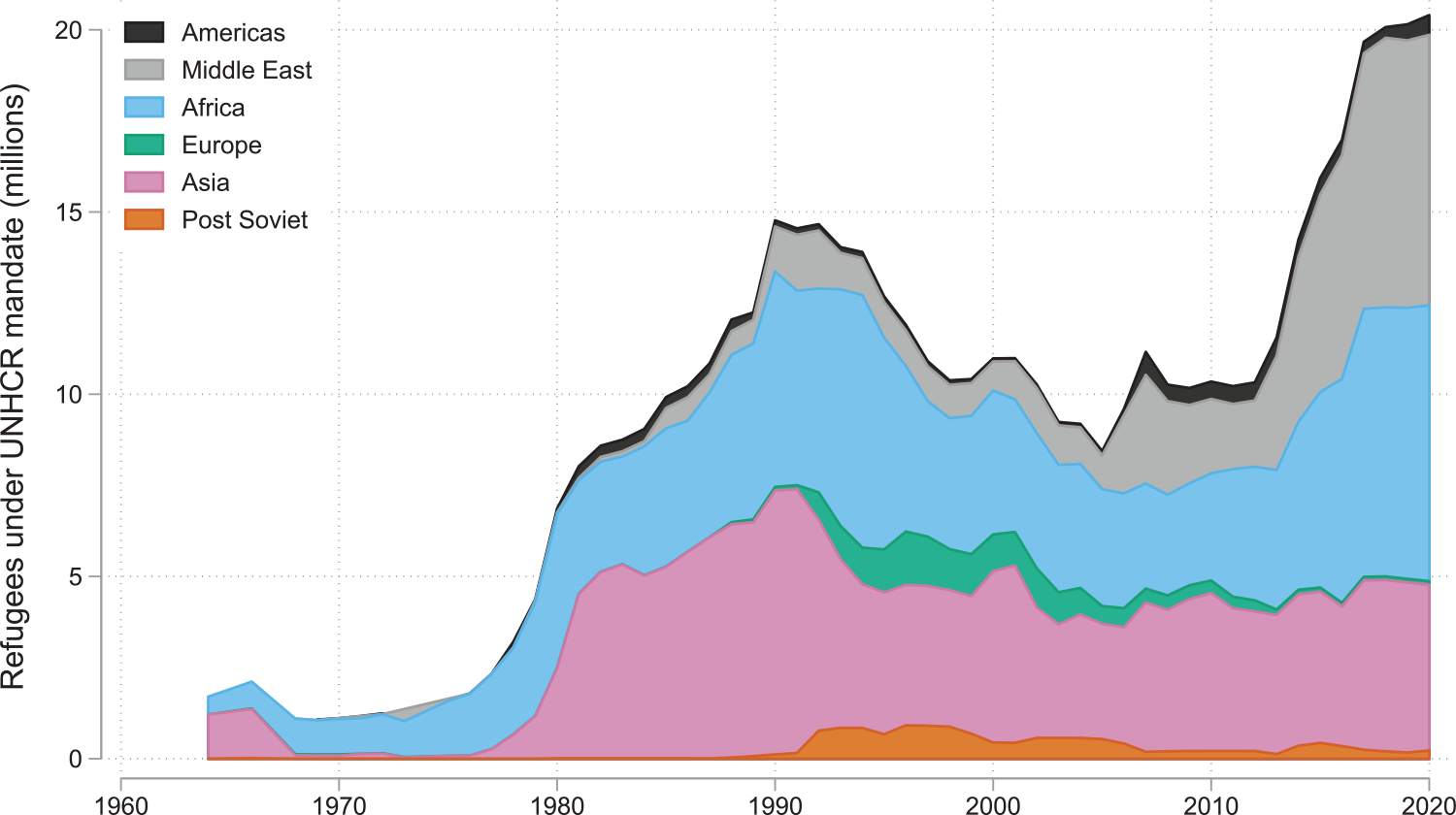
Refugees under UNHCR mandate, by region of origin. *Notes*: The data are from the UNHCR and available to download at https://www.unhcr.org/refugee-statistics/download.

Figure 1 plots the total number of refugees globally between 1960 and 2020 by origin region, as enumerated by the UNHCR. Recent years have witnessed unprecedented numbers of refugees across the globe, notably from Afghanistan, Myanmar, South Sudan, Syria, and Venezuela, which in tandem with the availability of data have spurred a renewed focus on refugees.^8^

**Figure 2:**
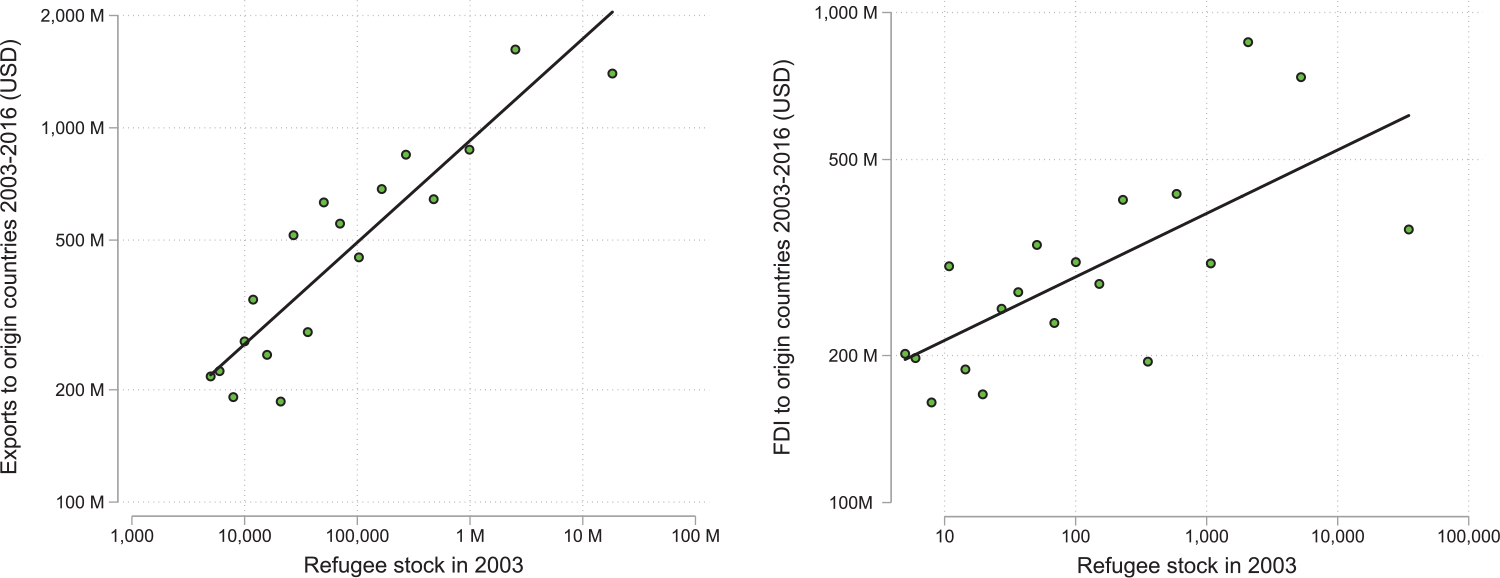
Exports and FDI outflows vs refugees. *Notes*: The figures are binned scatter plots of (*a*) exports and (*b*) FDI outflows during 2003–16 from each country to the refugees’ origin countries against the size of their refugee diasporas in 2003. *Sources*: UNHCR, COMTRADE, and fDiMarkets.

The binned scatterplots in Figure 2 present correlations of the positive association between refugee populations in 2003 and foreign direct investment (FDI; left panel) and exports (right panel) to refugees’ countries of origin in the following 15-year period. These relationships are robust to estimating gravity equations including origin and destination fixed effects, such that exports, FDI, and refugee populations are all expressed relative to the respective origin and destination averages.^9^ Figure 2 does not imply causation, however. This remains an enduring theme of the contemporary literature, which to date has relied most heavily upon natural experiments.

The current study focuses on refugees’ contributions to their home economies through the mechanisms of trade and FDI, under what circumstances these are made possible, and how governments might best engage refugee diasporas in this regard. First, we identify how refugees might differ from migrants on aggregate, while reviewing the extant literature. We then discuss the underlying mechanisms at play. In light of recent contributions, we subsequently delve into the role refugees can play in post-conflict reconstruction. Finally, we discuss policies, specifically how governments can best leverage overseas diasporas for the sake of economic development.

## II. Links between refugees, trade, and FDI

### (i) Refugees as a subset of migrants

As Gould (1994) first noted, migrants are necessarily different to natives and through their maintaining their ‘links’ to their home country they are able to foster international (trade) flows. Refugees, a subgroup of migrants, are essentially different from migrants, although the lines between them sometimes becoming blurred (Van Hear, 2005).^10^ While (economic, family, etc.) migrants have agency as to when and where they will move, those fleeing persecution rather represent forced migrants.^11^ Refugees’ immediate destinations are also typically reduced to a limited set, likely determined by perceived levels of safety. Refugees also often differ from migrants more broadly in terms of their origin countries. As such, they may represent new populations in host countries.

In seeking sanctuary, refugees often languish for years in camps. Indeed, even for those fortunate enough to be resettled, delays are common. The ultimate timings of refugee resettlements are therefore often shrouded in uncertainty (Beaman, 2012). So too are refugees likely selected on both observable and unobservable traits. As Mayda *et al.* (2022) note, for example, of those refugees deemed ‘most vulnerable’ by the UNHCR—those therefore deemed suitable for resettlement—less than 1 per cent are actually resettled in the United States.

Refugees’ journeys themselves also likely result in various forms of selection—not least since it is natural to think that refugee characteristics will differ depending on the timing of their departure. Those most able to depart will likely emigrate first. Refugees, if subsequently resettled, also often have little or no agency to decide the location where they will initially settle.^12^ Refugees’ ultimate success will likely therefore be a function of a combination of their personal characteristics, the circumstances of their departure, and the ultimate location of resettlement.

A general inability to pre-plan their departures, for example, often results in refugees being unable to take their capital with them; while their inability to return to their origins, at least initially, results in refugees having different incentives to invest (in human capital) at their destination (Cortes, 2004). Becker *et al*. (2020) for example, show that Polish descendants of those forcibly displaced from Eastern to Western Poland are significantly more educated today. Murard and Sakalli (2018) rather find that Greek locales hosting greater shares of refugees in 1923 enjoy greater levels of educational attainment today. Toews and Vézina (2021) in the Russian context, argue that the descendants of the Enemies of the People, the educated elite sent to the Gulag during Stalin’s reign of terror, are more likely to be college educated today. Chand and Clemens (2019), in the case of Fiji, provide evidence that the Indian persecuted minority invest more in their own human capital—which is mobile—given the increased likelihood of needing to exit the country. Most recently, Aksoy and Poutvaara (2021) show that refugees across Europe in 2015 and 2016 are positively selected on human capital. This evidence is consistent with the theoretical models by Katz and Rapoport (2005) and Poutvaara (2008).

All else equal, higher levels of human capital are likely to be associated with greater trade and investment volumes. The regression coefficients in column (3) of the Appendix Tables 1 and 2, however, rather suggest that refugees on average exert less influence when compared to non-refugee migrants. One plausible candidate to explain this result would be under-performing or non-functioning home markets, an explanation that we examine in more detail in section IV.

### (ii) Refugees, trade, and FDI

Of the fairly limited literature that examines the links between refugees and trade or FDI, eight pertain to trade, while only two feature FDI. Please refer to Table 3 in the Appendix for a tabular overview. Turning to the former first, the pioneering study by Head and Ries (1998) found little support for the conjecture that refugees foster international trade flows in the case of Canada. White and Tadesse (2010) argue in the context of trade with the US that refugees’ preferences may be skewed by the protracted lengths of time spent in third countries and, moreover, that their networks could have been eroded at origin if members of their network face persecution. Both migrants and refugees are argued to offset the trade-attenuating effects of cultural distance, with this latter effect reported as being definitively higher for refugees. One potential explanation would be the fact that refugees often represent new populations in their host countries. While contemporary advances in gravity models are absent (Anderson and Wincoop, 2003; Silva and Tenreyro, 2006), these authors nevertheless laid the conceptual foundation for the subsequent economics literature.

**Figure 3:**
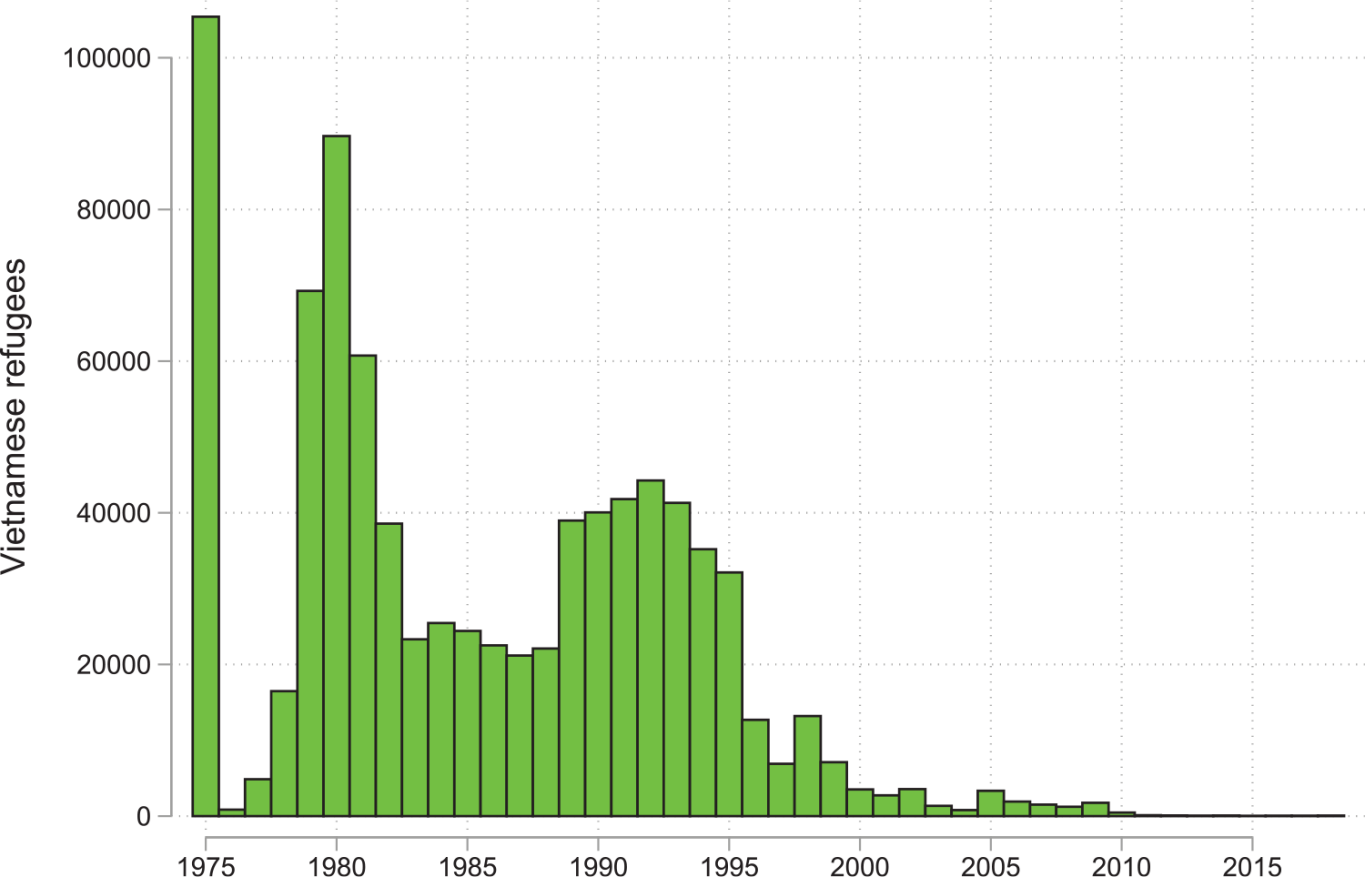
Vietnamese refugees and FDI to Vietnam. *Notes*: The export data are from United States International Trade Commission (USITC) and the refugee data from Parsons and Vézina (2018) and Dreher *et al.* (2020).

Two more recent offerings focus on the US, which reflects the fact that the US refugee data have been made more readily available to researchers.^13^ Parsons and Vézina (2018) exploit the natural experiment of the dispersal of the first wave of Vietnamese Boat People to enter the US, an event that occurred during a complete trade embargo on Vietnam. The first wave of Vietnamese refugees to the US occurred after the Fall of Saigon in 1975 when North Vietnam invaded in the South and put an end to the US involvement in the Vietnam War. This first wave was resettled across US states by voluntary agencies who made sure the new arrivals would be dispersed across the country so as not to repeat the experience of Cubans in Miami. In the following 20 years, around 1.4 million Vietnamese refugees were resettled in the US (Figure 3) and the Vietnamese became the largest refugee population in the US until ties between the US and Vietnam were restored. US exports to Vietnam over the period 1995–2010, following the lifting of the trade embargo in 1994, grew most in those US states with larger Vietnamese populations, themselves the result of larger refugee inflows two decades earlier. Elasticities of state exports to Vietnamese refugees range between 0.45 to 1.4 (Figure 3). One globally recognized product that resulted was Sriracha chilli sauce, produced by Huy Fong Foods in California. This company was founded by David Tran, who fled from Vietnam in 1979 following the Sino-Vietnamese war, naming his company after the Taiwanese freighter that ultimately delivered him to sanctuary.

Steingress (2018) differs in terms of leveraging variation in refugee allocations across origin countries and US states, while exploiting the dispersal of US refugees without ‘US ties’ for the sake of causal identification. Notably the settlement locations of refugees without US ties are chosen by the voluntary organizations as opposed to by the refugees themselves. Steingress (2018) estimates an elasticity of exports to immigrants (across US states and origin countries) of around 0.1. This is of a similar magnitude (0.15) reported in a meta-analysis of the broader trade and migration literature (Genc *et al.*, 2012).

Cucu and Panon (2018) motivate their cross-country study of EU asylum policies and trade by arguing that diplomatic relationships that shape asylum policies with rival countries may explain the lower observed effect of refugees in early studies. Adopting a saturated structural gravity model, the authors’ results suggest that a 10 per cent increase in refugee recognition rates—notably as opposed to actual refugee numbers—are associated with a 1.1 per cent fall in EU imports from refugees’ origin countries. No such evidence is found for EU exports. This is a novel finding since the economics literature has traditionally focused upon the influence of immigrants on exports. This is because any effect then necessarily operates in the opposite direction to immigrants’ preferences (see Parsons and Winters (2014)).

Two papers examine the trade-creating role of refugees based in camps. The sole time series paper in the literature, Ghosh and Enami (2015), examines the case of Afghan refugees settled in Pakistani refugee camps. They argue that the number of refugees does not ‘Granger cause’ bilateral trade flows. Since the countries border one another, both historically constituting part of the Silk Road, not only are the two countries more culturally similar, but so too are they likely to have traded with one another for centuries. Since Afghans in Pakistan number around 1.4 million, it is likely that some of the opportunities for trade may have already been exhausted. An alternative explanation is that refugees in these particular camps do not have the same incentives or the means to foster trade or FDI; or that trade or investment that does occur is not suitably recorded. Taylor *et al.* (2016) rather study three Congolese refugee camps based in Rwanda. They construct a general equilibrium model based on locally collected microsurvey data. Monte Carlo simulations yield an estimate of an increase of $49 in trade between a camp and the rest of the country *per* refugee hosted *per* year.

**Figure 4:**
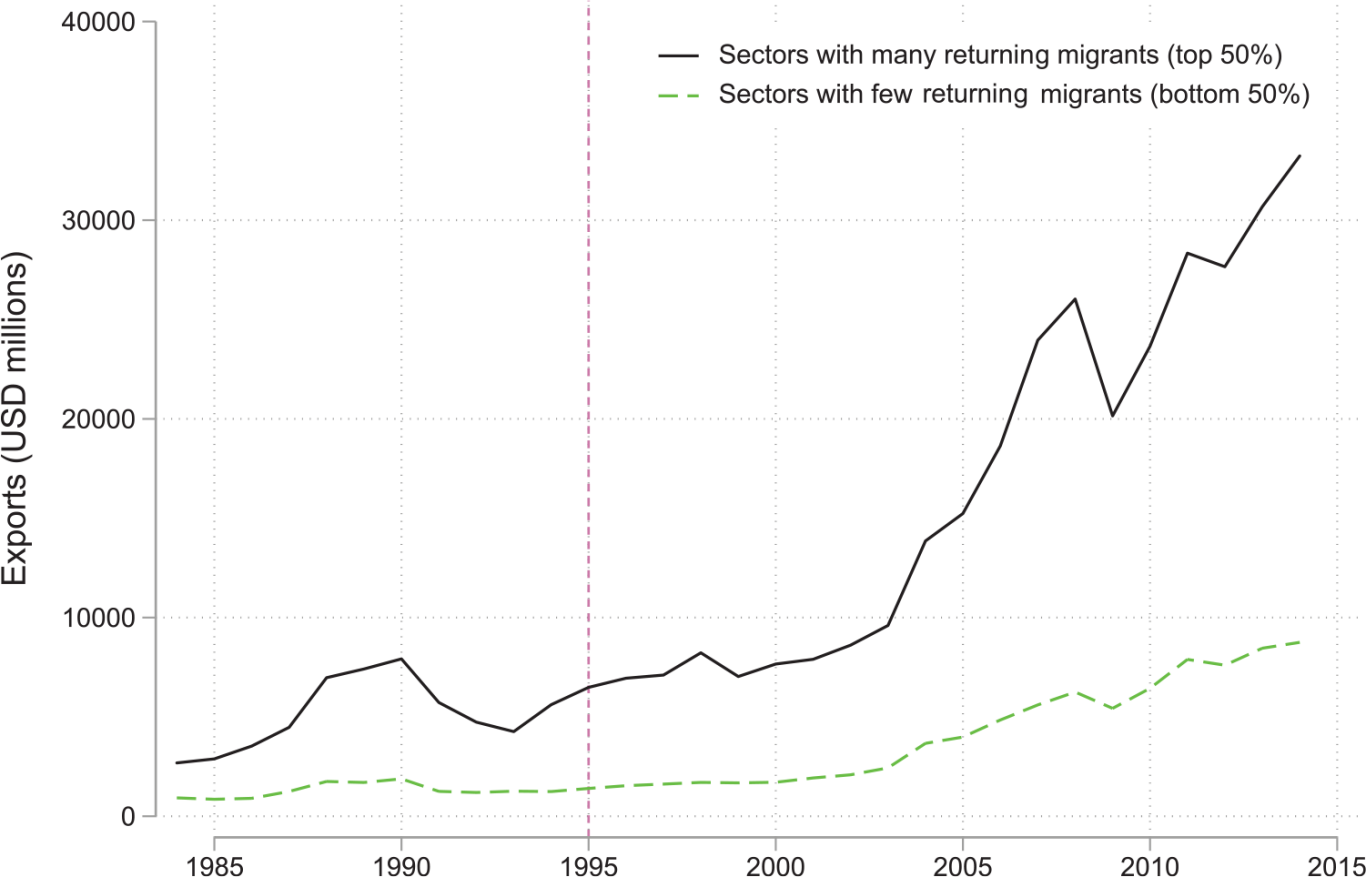
Exports from the former Yugoslavia for products with different levels of return migration. *Notes*: The figure plots the cumulative value of exports from the former Yugoslavia to the rest of the world across years. The two groups of sectors are defined by the number of returning migrants from Germany by 2000.

Bahar *et al.* (2022) notably differs from the remainder of the literature in investigating the role of Yugoslav refugees *returning* from Germany on Yugoslav exports to the rest of the world. Germany offered temporary protection to over 700,000 Yugoslavian refugees fleeing civil war during the early 1990s. By 2000, many had been repatriated. Exploiting confidential social security data capturing the employment of Yugoslav refugees in over 700 tradable German industries, those authors find that former Yugoslavian nations’ industries performed better, as measured by exports and labour productivity, the greater the number of returnees employed in those industries. This is illustrated in Figure 4, which shows that Yugoslav exports boomed in those sectors that witnessed larger numbers of returnees. Refugees in this instance notably increased *global* Yugoslav exports; an important contribution since the effects are shown to take place beyond refugees’ origin and host nations.

The literature examining the links between refugees and FDI is even more limited, primarily due to the dearth of FDI data available. Such time series typically begin in the early 2000s, with the exception of the data provided by the OECD.^14^ Indeed, the two most recently published papers on the broader topic subject (Burchardi and Hassan, 2013; Mayda *et al.*, 2022) rather employ costly privately compiled data.^15^

Burchardi and Hassan (2013) examine the role of ethnic ties and social capital in the context of ethnic German (*volksdeutsche*) and German citizen (*reichsdeutsche*) expellees (*vertriebene*) that subsequently resettled in West Germany. They find that firms based in districts in West Germany where expellees represented a larger share of the population in 1989 were more likely to operate a subsidiary or a branch in East Germany in 2007. They suggest that expellees thus fostered investment links with their regions of origin.

**Figure 5:**
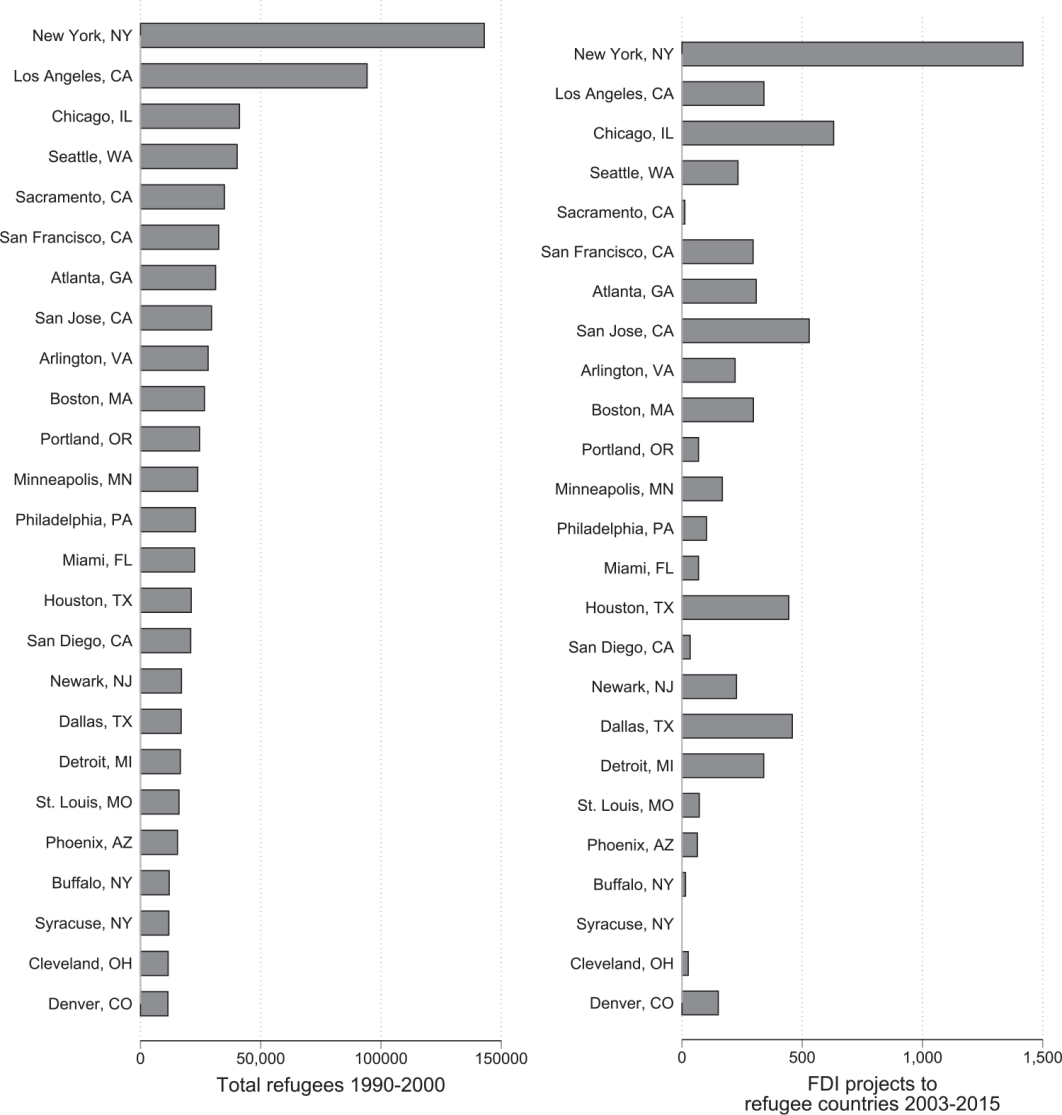
Refugees and FDI. *Notes*: The data are sourced from fDiMarkets and Dreher *et al.* (2020).

Mayda *et al.* (2022) combine the identification strategies of Parsons and Vézina (2018) and Steingress (2018) in the context of trade, to instead examine the links between refugees and FDI. In their primary analysis, they explore this link across US commuting zones leveraging US refugees without ties for causal identification. In their secondary analysis, they rather return to the natural experiment of the initial settlement of the Vietnamese in 1975 so as to further explore the underlying mechanisms and the role of the government of Vietnam’s policies enacted to foster FDI to Vietnam. Both identification strategies, which also control for city size and economic openness, suggest a causal effect of hosting refugees on fostering FDI to their origin countries. The authors find that a 10 per cent increase in refugees in a US city increases outward FDI flows to their countries of origin by 0.54 per cent. These patterns are illustrated in Figure 5. American cities hosting the largest numbers of refugees are concurrently the main sources of FDI to those refugees’ countries of origin.

## III. Mechanisms

The mechanisms governing the links between migrants (refugees) and FDI broadly mirror those underpinning the links between migrants (refugees) and trade (Leblang, 2010). The modern literature, diametrically opposed to the seminal contribution of Mundell (1957), offers both theoretical and empirical evidence in favour of migration and trade being complements. The catalyst for this now considerable literature began with seminal paper of Milgrom *et al.* (1990) who established the role of the Lex Mercatoria, or the Merchant Law enforcement system, as an institution that coordinated the role of agents in environments characterized by limited knowledge and trust. Continuing in this tradition, Avner Greif further examined the trade-promoting roles of Maghribi Traders’ Coalition in the eleventh century and the merchant guild during the Commercial Revolution of the eleventh to fourteenth centuries (Greif, 1989, 1993; Greif *et al.*, 1994). In doing so, Greif established that social institutions may underpin trading relationships, quite apart from established and competing theories, namely endowments, technology, and preferences (Greif, 1992). By disseminating information about members’ past actions and by coordinating appropriate responses, social institutions may serve as commitment enforcement mechanisms that ensure continued cooperation between trading members.

The trajectory of the empirical literature has largely paralleled data availability, such that the FDI literature lags the trade literature by almost two decades. The fundamental insight at the heart of the first empirical trade study (Gould, 1994), is that while migrants and natives constitute fundamentally disparate groups, immigrants maintain links with their home nations. Given the nature of refugee departures, however, which often occur against a backdrop of conflict and persecution, *a priori* one might think refugees sever their home ties. This is akin to the network degradation argument of White and Tadesse (2010). The weight of evidence, however, rather suggests that refugees indeed maintain strong links with their home nations, an aspect we examine in greater detail in section IV.

Gould distinguishes between two underlying hypotheses through which immigrants might foster trade. First, the preference hypothesis pertains to the local demand for products at origin, which is subsequently transplanted to destination once migration has occurred. Take the sale of khat, a stimulant used throughout (the Horn of) Africa and the Middle East, for example. Khat, while banned in every other developed country, was sold in London markets, thereby serving refugee communities—not least Somalis and Yeminis—until its eventual ban in 2014 (Kassim *et al.*, 2015). While it is fairly certain that such transactions of cultural goods would not have occurred in the absence of migration, it is also likely that these sales would have taken place at origin had no migration been observed. What might differ, therefore, are the prices charged or the surpluses captured, but no trade creation will have necessarily occurred, although variety at destination likely diversified.

Contrastingly, Gould’s immigrant-link hypothesis operates through migrants lowering the transaction costs of trade, which necessarily leads to trade being created. Migrants lower costs by more easily communicating with their compatriots, obtaining superior foreign market information, and negotiating and enforcing contracts with fewer impediments—often by relying upon their social networks. The theoretical and empirical literatures were subsequently advanced by James Rauch, whose name is synonymous with the ‘network/search view of trade’. The crux of Rauch’s arguments is that the heterogeneity in traded manufactures along ‘the dimensions of both characteristics and quality’ (Rauch, 1996) leads to prices no longer reflecting sufficient information to allow them to be transacted on international organized exchanges (Rauch,1999).^16^ Since buyers and sellers are rather matched through a costly process, which is a function of both the proximity between agents as well as ‘pre-existing ties’, Rauch—drawing on Granovetter (1973)—argues that weaker ties might yield superior outcomes since available opportunities are likely inversely proportional to the overlap between the information sets of the two parties. Rauch therefore argues that migrant (or refugee) networks foster the trade of differentiated as opposed to homogeneous goods (Casella and Rauch, 2002). This argument has since found empirical support, not least in the limited literature on refugees and trade (Parsons and Vézina, 2018; Steingress, 2018). While Greif emphasizes the role of networks in constituting social institutions that provide information about agents’ past behaviour, therefore, Gould and Rauch rather underscore the roles of immigrant-links and migrant networks in overcoming issues resulting from incomplete information.

Understanding the implications of these underlying mechanisms in turn proves instructive as to how these links might differ between migrants and refugees. Gould’s immigrant-link hypothesis suggests that migrants’ ability to stimulate trade is a function of migrants’ ‘existing foreign market information’ in tandem with their ability to integrate into their host-country communities (Gould, 1994). A priori therefore, it is not unreasonable to assume that refugees with better education and greater knowledge of home markets—those, for example, employed in relevant industries—would exert more influence on trade and FDI flows. In the original study, however, Gould actually finds that more-educated migrants are associated with lower trade flows.^17^ The conclusion is that while all immigrants lower the transaction costs of trade, more-educated migrants are more likely to create import-substituting businesses. This argument was first advanced in the context of the Argentine Republic (Díaz-Alejandro, 1970).

Whereas refugees’ skills have not been the focus of the associated literature, Mayda *et al.* (2022) provide some evidence that former (i.e. before resettlement) white-collar workers exert greater effects on FDI relative to blue-collar workers. This is especially true in skill-intensive FDI sectors: Software and IT Services, relative to Consumer Products, for example. Similarly, Bahar *et al.* (2022) demonstrate that returning educated Yugoslav refugees, and those engaged in occupations more apt to transfer knowledge, play a pivotal role in fostering Yugoslav exports relative to their less educated compatriots. These findings are broadly in line with the sister literature on trade and migration (Herander and Saavedra, 2005; Felbermayr and Toubal, 2012; Sangita, 2013; Aleksynska and Peri, 2014).

**Figure 6:**
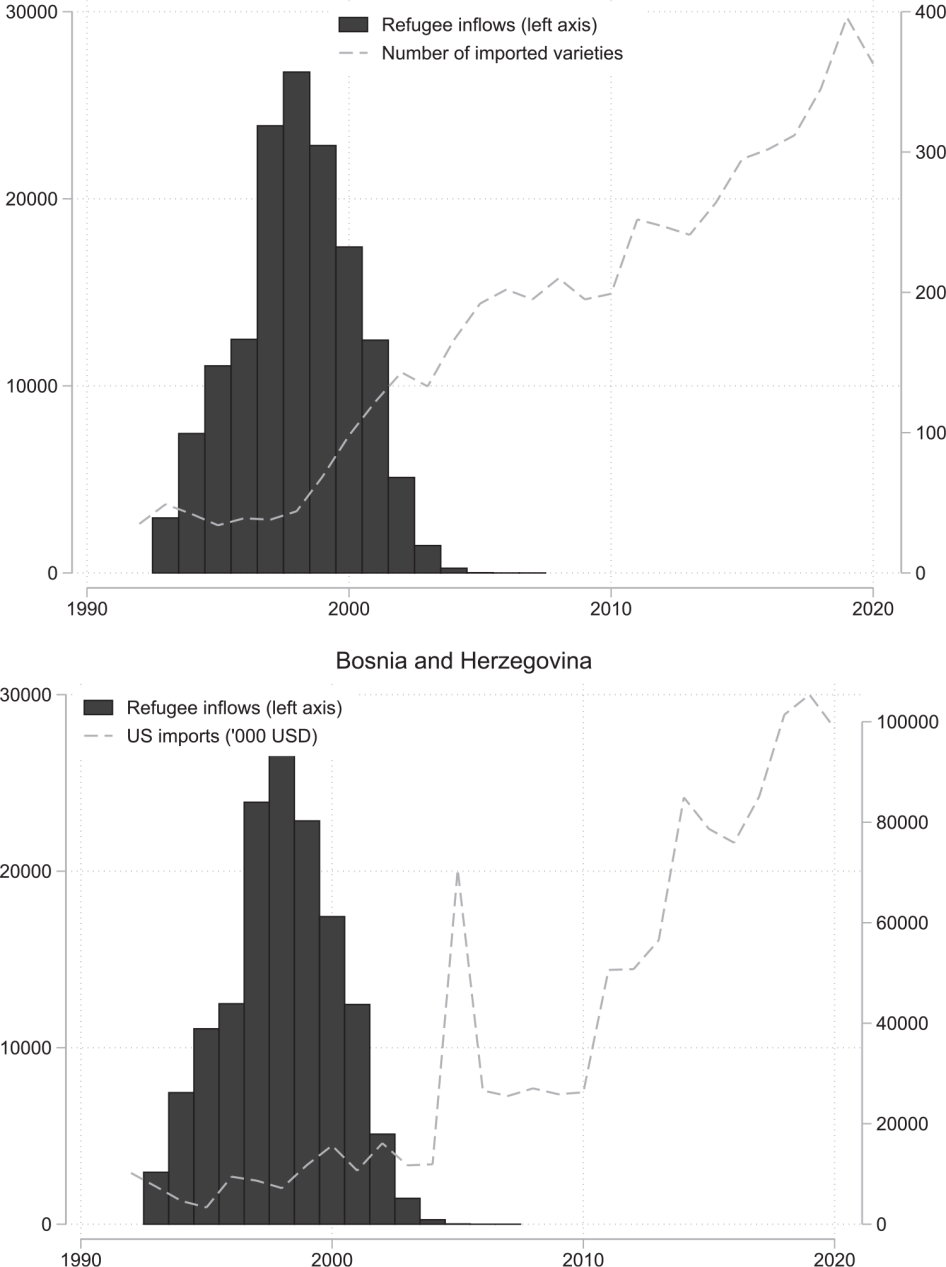
Refugees and imports from Bosnia and Herzegovina. *Notes*: The data are from UN Comtrade (HS 6 digit product categories) and Dreher *et al.* (2020).

A defining feature of refugee waves is that they often constitute new populations in their host countries. In the case of the US, for example, only relatively few Burmese, Ethiopian, or Vietnamese (students) resided within her borders prior to the US hosting major refugee waves from those countries. In the absence of sufficient foreign market information, therefore, incoming refugees can represent serious opportunities for traders and investors to break into new markets.^18^ Even should some level of trade or FDI occur in the absence of refugees, their addition likely yields new opportunities becoming available. In the case of Bosnian refugees, for example, trade between Bosnia and the US was previously limited to a narrow range of products. Following the Yugoslav wars of the 1990s, US imports from Bosnia diversified significantly, increasing from about 50 products at the end of the 1990s to more than 200 by the end of the 2000s; see Figure 6.

In this sense, refugees serve as conduits into new markets as new market opportunities are made available through their migration. A useful, although simplifying, heuristic is to consider a finite opportunity set for each newly established refugee corridor. In this vein, both Gould and Rauch uncover diminishing returns to network size.^19^ Similarly, Gould’s estimates of migrants’ duration of stay, suggest a waning of home-preferences over time, while, conversely, opportunities for exports increase after around 4 years. These results might be indicative of the process of assimilation.

In countries of refugee resettlement, refugees also often hail from non-aligned and culturally dissimilar countries. These facts could explain why refugees might actually exert an even greater effect on trade and FDI as when compared with migrants more broadly (should, indeed, trading and investment opportunities be inversely proportional to the overlap between the information sets of natives and incoming refugees). The existing evidence base, however, is insufficient to draw firm conclusions at this juncture. Indeed, some of the evidence presented in this paper provides reason to think the opposite could hold true.

Any observed effects could be larger in environments characterized by weak institutions, in which contract enforcement is more costly and trusted networks more valuable. In other words, the effects of refugees could be larger on trade with and between developing countries, especially least developed countries (Anderson and Marcouiller, 2002; Berkowitz *et al.*, 2006). Weak institutions have also been proffered as a potential explanation of the Lucas Paradox according to which capital does not flow from rich to poor countries (Lucas, 1990). The fact that refugees may foster FDI (and trade) to their origins, provides one mechanism through which refugees can make capital flow to poor countries. In that sense, refugees have the potential to meaningfully contribute to the economic development and potential reconstruction of their home countries.

## IV. Refugee diasporas and post-conflict reconstruction

**Figure 7:**
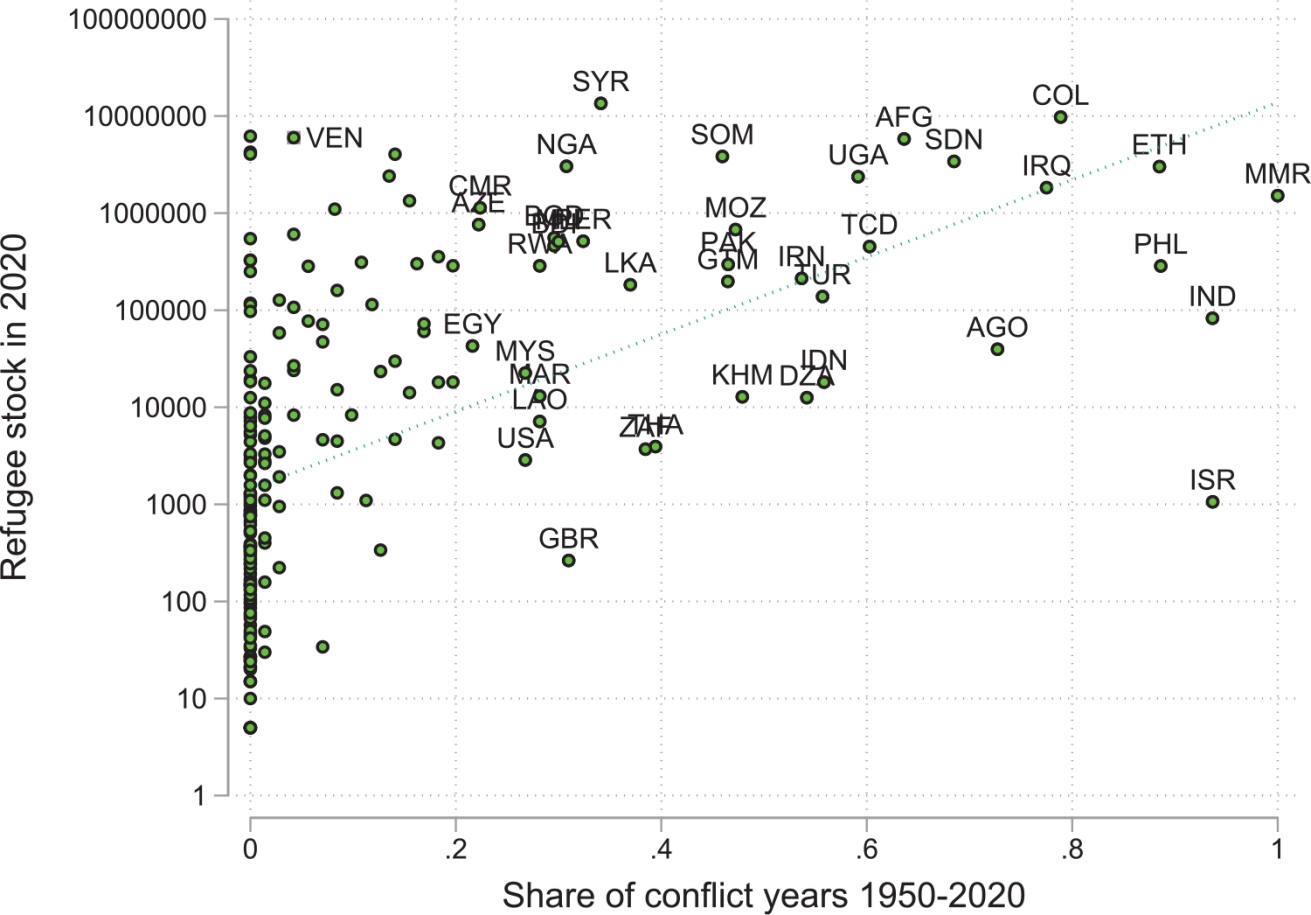
Conflicts and refugees outflows. *Notes*: The refugee stock variable captures the global stock of refugees by origin and is from the UNHCR. The *conflict* variable captures the share of years with a conflict during 1950–2020, based on the UCDP/PRIO Armed Conflict Dataset version 21.1.

Given the nature of their departure, refugee diasporas are intrinsically linked to the conflict they left.^20,21^ Figure 7 visualizes this relationship. It plots the log of total refugee diasporas by country of origin against the proportions of all years between 1950 to 2020 that those countries have been in conflict. The figure shows a clear positive correlation since longer conflicts tend to create larger refugee diasporas. Larger diasporas likely more easily marshal suitable resources together to aid the development of their origins. This is particularly likely to be true for resettled refugees, those typically relocated to countries far richer than their origins. The longer conflict endures, however, the longer ties have to erode.

It is somewhat intuitive, therefore, to consider that ties with the remaining populace might be severed, not least since Becker and Ferrara (2019) show that forced migration episodes can yield negative effects on those remaining. The available evidence however, suggests the opposite holds true. Mayda *et al.* (2022), for example, document the maintaining of ties in the case of the Vietnamese Boat People. Many ‘refugees rebuilt overseas networks with families and friends [and] Letters frequently moved between the receiving countries and Vietnam... (Zhou and Bankston, 1998)’.^22^ To this day, the flag of the former Southern Vietnam still flies above Little Saigon in Orange County.

**Figure 8:**
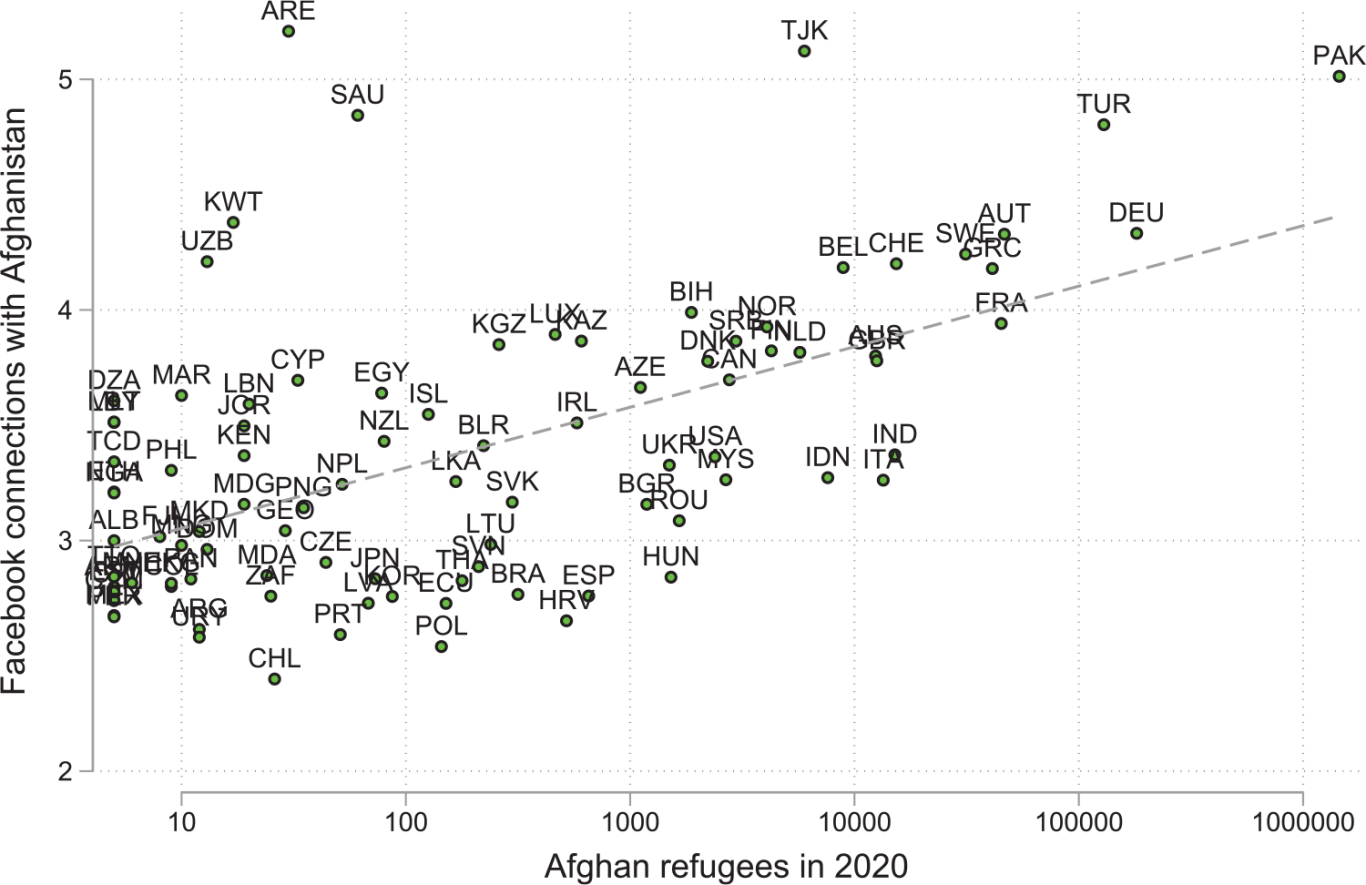
Facebook connections vs refugees. *Notes*: The figure plots Facebook’s social connected index in 2021 from each country to the refugees’ origin countries against the size of their refugee diasporas in 2020. The data are transformed using a log and are relative to importer and exporter means. *Sources*: HDX OCHA Services, and UNHCR.

Figure 8 complements these findings by plotting the relative number of Afghan refugees hosted by countries worldwide against the reported number of Facebook connections. Despite the fraught circumstances of many of their departures, a clear positive correlation results. Countries that host larger populations of Afghan refugees have more Facebook connections in Afghanistan. Indeed, many of the Afghan national cricket team honed their craft in Pakistani refugee camps.

The establishment and maintenance of contact between refugee diasporas and those remaining behind, doesn’t guarantee the diasporas’ intentions are pro-development—especially if selected on political traits. Former Cuban refugees in Florida, for example, were notoriously anti-Castro, lobbying against the removal of US sanctions. To this day Radio Erena in Paris, together with Agenzia Habeshia in Italy, both aim to broadcast widely the conditions that those in Eritrea face. Trade and investment can also be wielded along these lines. Campbell (2012), for example, argues that diamonds from Sierra Leone helped bankroll both the Lebanese civil war and the events of 9/11, although this remains conjectural.

**Figure 9:**
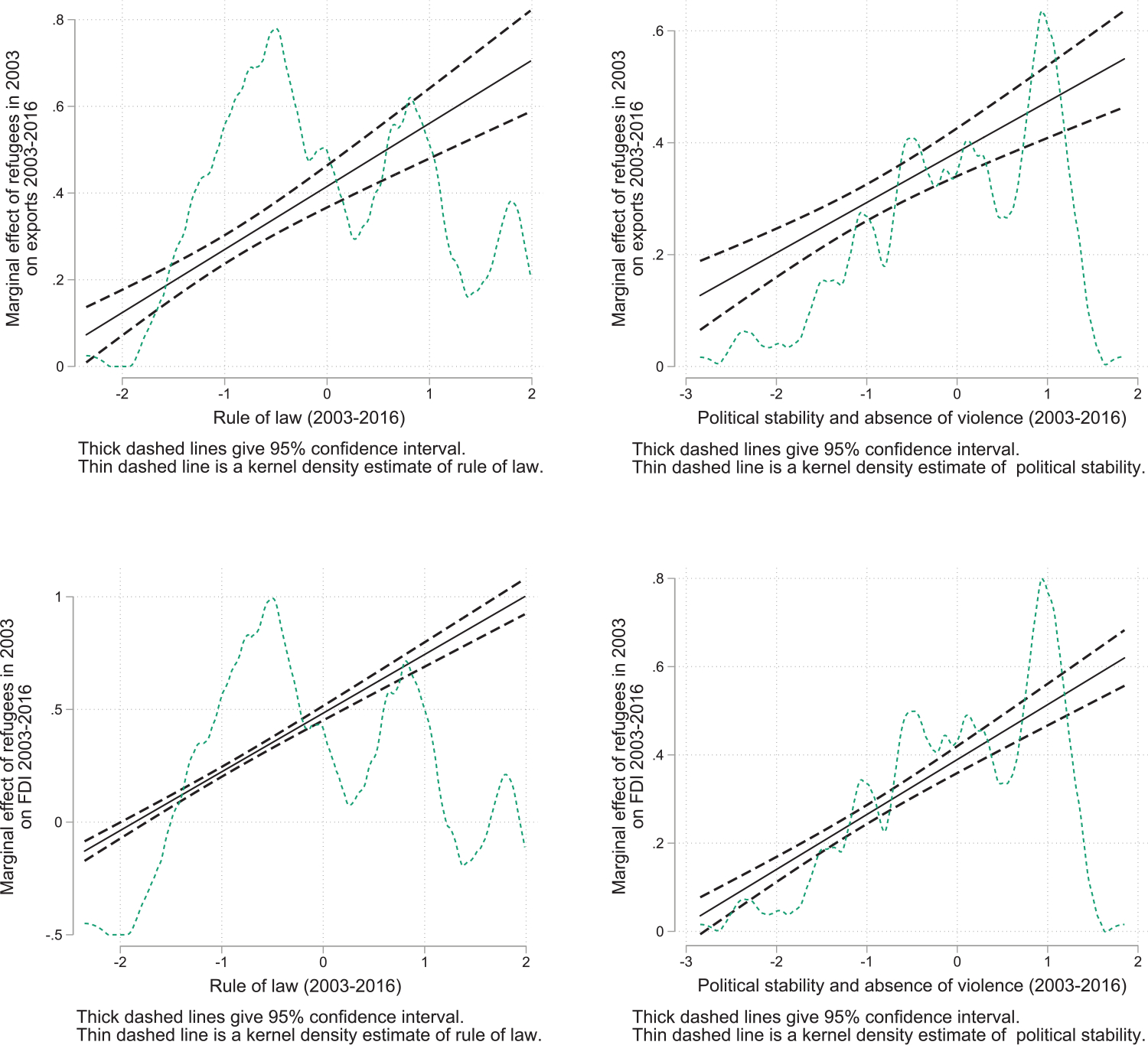
The effect of refugees on exports and FDI: the role of rule of law and political stability in origin countries. *Notes*: The figures plot the effect of refugee stocks in 2003 on (*a*) exports and (*b*) FDI outflows during 2003–16 from each country to the refugees’ origin countries against the level of rule of law or political stability and absence of violence in origin countries. Refugees, exports, and FDI figures are transformed using the inverse asymptotic sine (asinh) and are relative to importer and exporter means. *Sources:* UNHCR, COMTRADE, fDiMarkets, and World Governance Indicators. The corresponding regression results can be found in columns 4–7 in Tables 1 and 2 in the Appendix.

A growing body of evidence rather supports a complementary story wherein refugees are able to contribute to the development of their home nations. New trading and investment opportunities can only be taken advantage of, however, if market opportunities exist at origin.^23^ Mayda *et al.* (2022) provide evidence showing that political stability and the establishment of the rule of law at origin foster the FDI-creating role of refugees. Evidence along this dimension is presented in Figure 9. This shows the attenuating effects of the absence of the rule of law, political instability, and violence on refugees’ ability to foster trade and FDI flows.^24^ Refugees, on average, exert no effect on trade or FDI whatsoever, should their origin countries suffer political instability or an absence of the rule of law. Subsequently, the figure demonstrates strong complementarities between formal institutions and refugee networks in fostering trade and FDI to their origin countries, should such measures of good governance sufficiently improve. At this juncture, we simply lack a suitable body of quantitative evidence to meaningfully evaluate the extent to which trade and FDI contribute to the economic growth of refugees’ origins, however.

Perhaps most visibly, refugees send significant remittances home (Lindley, 2009), although distinguishing these from migrants’ remittances more broadly remains challenging (Legrain, 2016). Some evidence even suggests that refugees remit more frequently than any other type of migrant (Hugo *et al.*, 2011), which is intuitive given the likely inaccessibility of capital at origin. Broadly, remittances have been associated with new venture funding as well as founding rates (Vaaler, 2011), while the last three decades have witnessed remittances constituting an increasingly important source of development finance (De Haas, 2005). Existing evidence certainly suggests that remittances play a starring role in fostering entrepreneurship in developing countries (Woodruff and Zenteno, 2007; Yang, 2011). Anecdotal evidence also suggests remittances play a pivotal role as development finance and in paving the way for future trade and investment. Farah (2009), for example, documents that the Somali diaspora contributed to the reconstruction of hospitals, schools, airfields, and business centres following the collapse of the state apparatus in 1991. The precise role that remittances play in fostering and facilitating FDI and trade flows catalysed by refugees remains largely unstudied, however.

## V. Refugee diaspora engagement policies

### (i) Developed country policies

Since around the turn of the century, migration and development has been cast into the limelight, whereas beforehand it was considered a marginal issue (Bakewell, 2009). The policies previously adopted by the Dutch government, for example, involved returning migrants in an attempt to leverage their skills at home for the sake of development. This was arguably a first attempt to combine migration policies with an international development strategy (Bonjour, 2005). Subsequently however, the Dutch pivoted to a ‘broader and more inclusive understanding of the relationship between migration and development’ (Sinatti, 2010). Concurrently, the international community became increasingly aware of the developmental potential of migration. Migration and development therefore emerged as a central theme at various high-level policy fora.^25^

2005 seemingly represents a watershed moment in this regard. In that year, the EU officially recognized diasporas as ‘actors of home country development’,^26^ while what emerged from the events before and around the 2006 UN General Assembly High-level Dialogue on International Migration and Development conceptualized migrants as ‘agents of development’ (Piper, 2009). Specifically, it is the transnational nature of refugees, their concurrent engagement with more than one economy, which has resulted in migrants and their diasporas being recognized as potentially important development actors (De Haas, 2006). Significantly less recognition has been given to any potential role of refugees’ diasporas, however. Indeed, many prior diaspora engagement policies fail to distinguish between refugee diasporas from those formed by non-forced migrants, therein failing to consider the inherent differences between the two groups, not least the circumstances of their departures and arrivals.

Certainly host nations—especially developed host nations—play a central role in providing suitable environments for refugees to thrive, regardless of which endeavour they might be pursuing. Given refugees are likely selected on a number of attributes, however, their initial location, which in turn will determine their opportunity set together with the composition of the local labour force, will likely be paramount in determining their economic success. These initial allocations are determined by the prevailing refugee allocation policy at the time. While different systems have not been the subject of academic enquiry *per se*, evidence nevertheless suggests that the networks into which refugees are placed play an important role (Martén *et al.*, 2019; Parsons *et al.*, 2020).

A recent and important body of work therefore serves to better match incoming refugees with available opportunities in local receiving economies (Bansak *et al.*, 2018; Trapp *et al.*, 2018). Perhaps most obviously, refugee employment bans necessarily preclude individuals meaningfully participating in *any* economic activity. Policies therefore designed to increase refugees’ labour market participation will likely not only result in superior labour market and integration outcomes (Marbach *et al.*, 2018; Fasani *et al.*, 2021), but also provide a suitable foundation for entrepreneurial refugees to establish businesses etc. Similarly, lengthy asylum processes have also been shown to stymie refugees’ employment chances (Hainmueller *et al.*, 2016). Indeed, a key finding resulting from a seminar on ‘Refugees as development actors’, one convened by the European Council on Refugees and Exiles (ECRE), the Danish Refugee Council, and Maastricht University in 2014, was that ‘[Refugees’] involvement in their countries of origin is in direct relationship with integration in the country of settlement’.^27^

The available evidence suggests that refugees’ remittances play a potentially important developmental role. Given the exorbitant fees charged by remittance providers, however, one low-hanging policy option would be to lower the associated transaction fees in an attempt to bolster refugees’ remittances (see, for example, Freund and Spatafora (2008)). Given the difficulties associated with delineating between the remittances of refugees and migrants more broadly, our evidence base could be improved with the advent of better recorded remittance flows. This issue speaks to the wider problem that migrants are typically recorded by their nationality, or their *country* of birth. Instead enumerating migrants and refugees and their remittances at *sub-national* geographical units would in turn likely spur significant new research in the migration and development sphere.

What has ultimately emerged on the ground in developed countries in response to increased refugee inflows, is a rich tapestry of developmental actors, including: government (ranging from local to federal), non-governmental organizations, civil society, and voluntary organizations. The DIASPEACE (Diasporas for Peace: Patterns, Trends and Potential of Long-distance Diaspora Involvement in Conflict Settings) project, funded by the European Commission, proves particularly instructive in this regard (Sinatti, 2010). The aim of the 3-year project was to examine how refugee diasporas might serve to foster peace and development at their origins. The project focused on diasporas originating from the Horn of Africa (Ethiopia, Eritrea, and Somalia), those residing in five European nations (Finland, Germany, Italy, Netherlands, and Norway). Field research was conducted in both Europe and Africa and interested readers are referred to the detailed case studies available in Sinatti (2010).

Broadly, despite refugees being recognized as constituting a separate group to migrants, they nevertheless often fell under broader ‘migrant’ policies. Clear differences also emerge in terms of the policies enacted (advocacy and consultation, capacity building, and access to credit/finance) and their coherence at various levels of government across European nations. Various challenges also arose in terms of how best to identify the most suitable individuals to partner with, and discrimination resulting from providing funds to one group and not another. These issues are especially troubling in the case of particularly fractionalized refugee groups, the Somalis in Italy, for example. Proposed solutions include focusing upon specific developmental themes as opposed to providing assistance to specific groups in an attempt to depoliticize any underlying tensions. Similarly, a growing trend has been to form umbrella groups that comprise myriad disparate groups so as to minimize competition between rival (ethnic) groups within the same refugee diaspora.^28^

### (ii) Developing country policies

Keenly aware of the important role overseas communities are able to play in fostering economic development at origin, developing countries have increasingly established ‘diaspora institutions’, the number of which has grown from 10 in 1970 to 40 in the year 2000 (Gamlen, 2014). According to Gamlen (2014), Vietnam in this regard specifically engages its diaspora through ‘investment policies and lobby promotion’. Harding and Javorcik (2011) argue that such investment promotion efforts more broadly lead to increased FDI in countries in which ‘red tape and information asymmetries are likely to be severe’ i.e. refugees’ origins.

One straightforward policy—although one that doesn’t leverage the inherent transnational character of refugees—would be to encourage talented and knowledgeable citizens residing abroad to return home. Depending on the context, however, any such repatriations will likely be complicated by property rights issues and the scars of conflict (e.g. landmines) even if the conflict has officially ceased (Chimni, 2002). Housing, specifically, has been identified as a central issue ‘commonly understood as a key ingredient in sustainable return and reintegration’ (Loschmann *et al.*, 2015). In instances in which wealthier and more successful refugees are considering returning, refugees’ origin countries could provide various financial incentives, through, for example, a reduction, temporary or otherwise, in the personal income tax rate.

Refugees need not return in order to play starring roles in the development of their home countries, however. In Mayda *et al.* (2022), for example, we document the FDI-enhancing roles of policies enacted by the Vietnamese government in an attempt to better leverage the *Viet Kieu*, the overseas Vietnamese community, for the sake of Vietnam’s development. These included the 2005 Investment and Enterprise Laws, which provided tax incentives and rent exemptions for foreign investors, while fostering a nurturing investment environment.^29^ So, too, was the diaspora specifically targeted. The 2008 Nationality Law, for example, eased the administrative procedures for *Viet Kieu* investors, allowing them to regain Vietnamese citizenship, thereby facilitating the ease with which they could do business with Vietnam. The 2008 Vietnamese Government Action Plan also provided incentives to *Viet Kieu* investors in the form of reduced land rents, cheap loans, investment credit guarantees, corporate and personal income tax breaks, while also lowering tariffs on machinery imports. In Mayda *et al.* (2022) we provide evidence that these reforms, indeed, boosted trade and investment into Vietnam. The commuting zones that hosted larger concentrations of Vietnamese fostered larger volumes of FDI to Vietnam following the enactments of the laws. The magnitude of the increase in FDI between the pre- and post-reform period varies between 0 and 8 per cent from the commuting zones with the most Vietnamese refugees.

Another salient example of successful refugee diaspora-engaging policies is that of Armenia. This case differs significantly from that of Vietnam, given how long ago Armenians emigrated in the wake of the 1915 genocide. Nevertheless, the Armenian Diaspora, 1.4 million strong in the US, is heavily involved in Armenia’s development. As Atabekyan (2017) writes in an article for EVN, a magazine based in Yerevan, the diaspora constitutes the principal investor in the Armenian economy, especially so since the dissolution of the Soviet Union. This fact explains the establishment of the Ministry of Diaspora in 2008, which is tasked with nurturing diasporic links with Armenia, in addition to the establishment of a number of formal associations aimed at fostering business linkages, e.g. the American Chamber of Commerce in Armenia, the Canada–Armenian Business Council, the Pan-Armenian Lawyers Association, and the Pan-Armenian Association of Engineers and Architects.

An UNCTAD report on investment promotion strategies in Armenia (UNCTAD and Armenia, 2020) confirms that the Armenian Diaspora represents significant opportunities for investment. The report identifies 108 business leaders across 20 countries, therein highlighting potential sectors of investment, including: high-tech and innovation activities, pharmaceuticals, education, wine, food processing, and fashion. While no formal analysis has been conducted to evaluate the efficacy of these policies, anecdotal evidence, together with the Armenian government reports, nevertheless provide some evidence in favour of the positive developmental role played by the Armenian diaspora.

## VI. Conclusion

The recent surge in the number of refugees globally has naturally catalysed further research into this specific group, which is delineated by the circumstances of their departure and their subsequently being granted refugee status. An enduring theme over the centuries is the remarkable degree to which refugees have spread ideas across the globe. Humanitarian policies enacted to provide sanctuary to vulnerable and marginalized groups are, therefore, able to yield unexpected economic dividends. An important subset of these unexpected economic dividends are increased trade and FDI flows—margins along which refugees can foster development at their origins.

While the associated literature documenting such effects is growing, our understanding nevertheless remains quite limited. In this paper, we first summarize the extant literature focusing upon studies that aim to establish causal links between refugees, trade, and investment. We subsequently delve into the underlying mechanisms underpinning these relationships. On the one hand, refugees might be expected to exert more influence on trade and investment when compared to migrants more broadly, since they often represent new populations in host countries and have greater incentives to invest in human capital. What evidence does exist, however, is mixed. One explanation for this finding is the context of the country from which the refugees fled—political upheaval and an absence of the rule of law.

Nevertheless, refugee diasporas represent enormous potential to contribute to development and post-conflict reconstruction of their home countries. To this end, we examine how various policies adopted in developed receiving, and developing sending, countries, have been enacted to foster refugees’ economic dividends. These include: establishing regulations and policies that differentiate between refugees and migrants, providing full access to refugees to local labour markets, reducing the costs of remittance flows, leveraging diasporas through engaging them through coordinated tapestries of local service providers and governmental institutions, as well as providing suitable incentives to return home in post-conflict situations.

In conclusion, more research is needed to deepen our limited understanding of the potential developmental benefits of suitably engaging with refugee diasporas. The research currently in the public domain suggests that these gains may indeed be very large, but we know little about best practice or optimal policy-making. In part, research until now has been hampered by the unavailability of refugee-specific data. As refugee data availability improves, it is natural to consider that researchers will understand these issues better in the coming years.

Finally, it is high time for the narrative on policies regarding refugees to be updated. Public policy discourse on refugees continues to focus almost exclusively on humanitarian issues, or the impact of refugees on host countries along a number of dimensions. It thereby often highlights the ‘costs’ involved. The evidence presented in this paper, however, draws a parallel and more positive narrative, one in which refugees and their associated diasporas represent serious opportunities—if accompanied by the right policies—to realize important economic gains that in the medium to long run might be expected to exceed any short-run costs of ‘accommodating’ them. In this sense, we hope that our study, together with our broader research agenda, will not only contribute to furthering our understanding on these topics, but, in tandem, will also serve as a vehicle to bridge the gap between the research and policy communities.

## Footnotes

1 https://www.jewishlivesproject.com/profiles/joseph-malin.

2 Which is also referred to as the Revocation of the Edict of Nantes.

3 https://www.rescue-uk.org/article/little-known-history-fish-and-chips.

4 Prominent examples in the case of the United States include: Madeleine Albright, Sergey Brin, Albert Einstein, Wyclef Jean, Steve Jobs, Thomas Mann, and Billy Wilder. In the UK, examples include: Joseph Conrad, Jacob Epstein, Victor Hugo, Lucien Freud, Sir Hans Krebs, Freddie Mercury, and Tom Stoppard.

5 https://www.refugeecouncil.org.au/refugees-are-entrepreneurial.

6 Indeed the Entrepreneurial Refugee Network (TERN) strives to establish 2,000 refugee-fronted businesses in the UK by 2025 (https://www.wearetern.org).

7 Betts *et al*. (2017), in examining data from five developed countries (Austria, Australia, Canada, Italy and the Netherlands), however, conclude that refugees typically operate low value-added businesses that subsequently employ few others.

8 Refer to Dustmann et al. (2017) and Becker and Ferrara (2019) for recent reviews.

9 Please refer to the Appendix for the corresponding regressions results in Tables 1 and 2.

10 This is despite the existence of distinct academic journals, research centres, and degree programmes that all focus upon refugees specifically.

11 While the 1951 Convention adopted a rather narrow definition of a refugee as one who fears persecution due to their race, religion, nationality, social group, or political affiliation; such a focus omits those fleeing widespread unrest, famine, economic collapse, and natural disasters.

12 Economists have therefore exploited refugee data for the sake of causal identification; see, for example, Edin *et al*. (2003), Damm and Dustmann (2014), Dustmann *et al*. (2019), Dreher *et al*. (2020), and Parsons *et al*. (2020).

13 See, for example, Dreher *et al*. (2020)://www.refugeeresettlementdata.com.

14 https://data.oecd.org/fdi/fdi-flows.htm.

15 ORBIS of Bureau van Dijk (https://www.bvdinfo.com/en-gb/our-products/data/international/orbis) and FDIMarkets (https://www.fdimarkets.com/).

16 Rauch and Casella (2003) extend the analysis to a more formal general equilibrium matching model, in which group-ties extend complete information about their domestic markets across international borders within groups, thereby fostering a more efficient international matching of pairs of producers.

17 With the exception of the coefficient for producer goods.

18 In the case of the Vietnamese in the US, for example, two decades of refugee resettlement occurred before the US considered lifting the trade embargo on Vietnam. By 1995, ‘US investors were champing at the bit’ (see Parsons and Vézina (2018)).

19 Gould estimates that around 12,000 immigrants exhaust around 90 per cent of the effect for aggregate exports flows.

20 Indeed, refugee repatriation is typically associated with the cessation of conflict (Chimni, 2002).

21 Whereas the U-shaped relationship between emigration and development has been the subject of significant study (see, for example, Clemens (2014) and Dao *et al.* (2018)), the relationship between conflict and violence and international emigration remain comparatively unstudied. Recent contributions include: Shellman and Stewart (2007), Bohra-Mishra and Massey (2011), Amuedo-Dorantes and Puttitanun (2016), Atuesta and Paredes (2016), Shrestha (2017), and Clemens (2021).

22 Indeed, Ho Thanh Viet who found refuge in California, actually developed the first software facilitating the electronic use of Vietnamese (Mayda *et al.,* 2022).

23 In the conventional sense, since civil war may itself present new opportunities. Blood diamonds, for example.

24 The corresponding regression results can be found in columns 4–7 in Tables 1 and 2 in the Appendix.

25 For example, Global Commission on International Migration in 2005, the UN High Level Dialogues on International Migration and Development in 2006 and 2013, the Global Forum on Migration and Development, the second UN Conference on Sustainable Development, Rio+20 in 2012, and the Millennium Development Goals Post-2015 development framework (Post-2015 Agenda).

26 See https://eur-lex.europa.eu/LexUriServ/LexUriServ.do?uri=COM:2005:0390:FIN:EN:PDF

27 https://ecre.org/wp-content/uploads/2015/01/DomAid_DiasporaPaper.pdf.

28 See for example: https://www.bond.org.uk/node/4455.

29 Harding and Javorcik (2011) show that investment promotion leads to increased FDI in developing countries.

## Appendix

**Table 1:** The effect of refugees in 2003 on FDI 2003–16

	(1)	(2)	(3)	(4)	(5)	(6)	(7)
	
	FDI	FDI	FDI	FDI	FDI	FDI	FDI

Migrant stock: 2000	0.235*** (0.017)		0.211*** (0.016)		0.205*** (0.015)		0.204*** (0.015)
Refugee stock: 2003		0.295*** (0.037)	0.174*** (0.037)	0.389*** (0.042)	0.272*** (0.041)	0.484*** (0.040)	0.367*** (0.039)
× Political stability				0.124*** (0.028)	0.118*** (0.027)		
× Rule of law						0.260*** (0.032)	0.253*** (0.030)
N	53664	53664	53664	47606	47606	47606	47606
R-sq	0.50	0.46	0.50	0.48	0.53	0.49	0.53

*Note*: Cross section with origin and destination fixed effects included in all regressions. Refugees are stocks in 2003. FDI is 2003–16. Standard errors in parenthesis are clustered by origin and destination, and

* stands for statistical significance at the 10% level

** at the 5% level, and

*** at the 1% level.

FDI, refugee, and migrant variables are in inverse-hyperbolic sines. *Political stability* and *Rule of law* are from the World Governance Indicators and we use the average of the indices for the years 2003–16. The coefficients suggest that both migrants and refugees have a positive effect on FDI, and that both these effects increase with the quality of governance. A 10 per cent increase in refugees from a particular origin is associated with a 2.95 per cent increase in FDI to that origin country.

**Table 2:** The effect of refugees in 2003 on exports 2003–16

	(1)	(2)	(3)	(4)	(5)	(6)	(7)
	
	Exports	Exports	Exports	Exports	Exports	Exports	Exports

Migrant stock: 2000	0.430*** (0.026)		0.419*** (0.026)		0.421*** (0.026)		0.421*** (0.026)
Refugee stock: 2003		0.309*** (0.054)	0.076 (0.050)	0.383*** (0.066)	0.164*** (0.049)	0.415*** (0.068)	0.189*** (0.055)
× Political stability				0.090 (0.060)	0.080 (0.058)		
× Rule of law						0.145* (0.075)	0.125* (0.074)
N	51076	58018	51076	50759	46104	50759	46104
R-sq	0.78	0.75	0.78	0.75	0.78	0.75	0.78

*Note*: Cross section with origin and destination fixed effects included in all regressions. Refugees are stocks in 2003. Exports are from 2003 to 2016. Standard errors in parenthesis are clustered by origin and destination, and

* stands for statistical significance at the 10% level

** at the 5% level and

*** at the 1% level.

Export, refugee, and migrant variables are in inverse-hyperbolic sines. *Political stability* and *Rule of law* are from the World Governance Indicators and we use the average of the indices for the years 2003–16. The coefficients suggest that both migrants and refugees have a positive effect on exports, and that both these effects increase with the quality of governance. A 10 per cent increase in refugees from a particular origin is associated with a 3.09 per cent increase in exports to that origin country.

**Table 3:** Papers on refugees and trade and investment

Paper	Context	Effects

Head and Ries (1998)	The effect of migrants and refugees on Canadian trade with 136 partners from 1980 to 1992	Refugees, unlike total migrants, have a negative effect on exports.
White and Tadesse (2010)	The effects of refugee and non-refugee immigrants on US trade with their home countries.	No effect of refugees except at large cultural distance
Burchardi and Hassan (2013)	East German expellees in West Germany foster investment in East Germany after re-unification	A 0.019 rise in the share of expellees (across German districts) is associated with a 2.8 percentage point increase in the probability of investment in the East
Ghosh and Enami (2015)	Afghan refugees in Pakistan	Afghani refugees do not cause trade between Afghanistan and Pakistan
Taylor *et al.* (2016)	Economic impact of Congolese refugees in Rwanda	Congolese refugees in Rwanda stimulated trade between the local economy and the rest of Rwanda by $55 per refugee per year
Cucu and Panon (2018)	Refugee admission rates in EU countries as a measure interstate tensions	Elasticity of EU imports to this refugee admission rates between 1.5 and 5.5
Parsons and Vézina (2018)	Vietnamese refugees in the US help US exports to Vietnam	Elasticity of exports to Vietnamese refugees between 0.45 to 1.4 (across US states)
Steingress (2018)	Refugees in the US are used as a instrument to estimate the effect of immigrants on trade	Elasticity of exports to immigrants around 0.1 (across US states and origin countries)
Mayda *et al.* (2022)	Refugees in the US foster FDI to origin countries	Elasticity of FDI outflows to Refugee inflows of .054 (across commuting zones and countries of origin)
Bahar *et al.* (2022)	Returning refugees to ex-Yugoslavia help boost exports	Elasticity of exports to return migration between 0.08 to 0.24 (across products)
